# Can We Still Trust Docking Results? An Extension of the Applicability of DockBench on PDBbind Database

**DOI:** 10.3390/ijms20143558

**Published:** 2019-07-20

**Authors:** Giovanni Bolcato, Alberto Cuzzolin, Maicol Bissaro, Stefano Moro, Mattia Sturlese

**Affiliations:** Molecular Modeling Section, Department of Pharmaceutical and Pharmacological Sciences, University of Padova, 35131 Padova, Italy

**Keywords:** molecular docking, docking benchmark, DockBench, virtual screening

## Abstract

The number of entries in the Protein Data Bank (PDB) has doubled in the last decade, and it has increased tenfold in the last twenty years. The availability of an ever-growing number of structures is having a huge impact on the Structure-Based Drug Discovery (SBDD), allowing investigation of new targets and giving the possibility to have multiple structures of the same macromolecule in a complex with different ligands. Such a large resource often implies the choice of the most suitable complex for molecular docking calculation, and this task is complicated by the plethora of possible posing and scoring function algorithms available, which may influence the quality of the outcomes. Here, we report a large benchmark performed on the PDBbind database containing more than four thousand entries and seventeen popular docking protocols. We found that, even in protein families wherein docking protocols generally showed acceptable results, certain ligand-protein complexes are poorly reproduced in the self-docking procedure. Such a trend in certain protein families is more pronounced, and this underlines the importance in identification of a suitable protein–ligand conformation coupled to a well-performing docking protocol.

## 1. Introduction

Since its introduction in the early 1980s [1], molecular docking has served to aid medicinal computational chemists in optimizing the drug discovery process. Ten years later, due to methodological and technological advances, together with the increasing number of experimentally solved macromolecular structures, it became possible to process more and more molecules within a docking procedure, opening the era of Structure-Based Virtual Screening (SBVS) as a strategy in selecting appropriate compounds from large virtual libraries on the basis of good protein–ligand interaction patterns [2]. Thanks to molecular docking, Structure-Based Drug Discovery (SBDD) field has become very popular today. A docking protocol can be described as the combination of a search algorithm that samples the conformational space of a ligand, generating conformations for the ligand itself (defined as poses) within a binding site, and a mathematical equation, called scoring function, which quantitatively evaluates the quality of such poses. The scoring function has always been the Achilles tendon of molecular docking due to the inaccuracy in quantified strength of the complex network of molecular interactions. Today, it is widely accepted that molecular docking has been outperformed by other structure-based in silico methodologies in investigating the stability and strength of the protein–ligand interaction [3], even though they are usually demanding techniques. However, molecular docking still represents a valid technique in sampling the conformations of the ligand in a binding site in a very efficient manner—at a fraction of the computational cost of more accurate methods based for example on Molecular Dynamics [4]. To prove the extensive adoption of molecular docking in research, there are more than 50 docking software options listed up to date in the on-line Click2Drug repository [5]. It should also be considered that each docking software usually provides more than one scoring function in which performance ought to be evaluated in the protocol tuning step. This means that computational chemists have at their disposal a plethora of different protocols when they face a docking calculation and, more importantly, the success, for example, of a Virtual Screening (VS) campaign, strongly relies on the accuracy of the protocol employed to place and rank the conformation of candidates into a target binding site [6]. To further complicate matters, additional considerations need to be taken into account. In fact, more and more experimental structures are thankfully available, hence the range of possible combinations in protein conformation-docking protocol is growing in an unstoppable trend. It is, therefore, clear that a crucial step in SBVS is the selection of a proper docking protocol and an appropriate protein conformation [7,8]. To address this issue, we recently proposed a platform, DockBench, with the aim of simplifying the non-trivial task of automatically comparing the performance of different docking protocols in a self-docking exercise. The criteria of selection of the most appropriate protocol are based on geometrical and statistical basis evaluating few observables: the lowest and the average Root Main Square Deviation (RMSD) obtained for a pose of the ligand compared to its crystallographic pose and the protocol score [9]. In 2011, Plewczynski et al. reported a comparison among seven docking protocols on the PDBbind (http://www.pdbbind.org.cn) that, at that time, counted on 1300 structures [8]. Here, we report a large benchmark of 17 different docking protocols compared on the basis of the self-docking procedure on a dataset of 4169 protein–ligand complexes. The notable number of structures has offered the opportunity to evaluate the performance of molecular docking from different points of view, underlining how the efficiency of docking protocols may vary depending on the nature of the protein family.

## 2. Results

The benchmark was performed on 4169 structures obtained from PDBbind, a free database of binding affinity data for biomolecular complexes including protein–ligand, nucleic acid-ligand, protein-nucleic acid, and protein–protein complexes [10]. The PDBbind “Refined set” is a subset of high-quality protein–ligand complex structures helpful for the validation of Docking protocols. All the structure needs to be processed prior to the docking calculation to keep only the protein and the ligand alone. This was necessary to simplify the execution on such a large set of complexes and protocols.

The preparation of the data was accomplished by an automatic procedure based on the Molecular Operating Environment (MOE) suite for proteins and OpenEye toolkit for ligands (vide infra, see method section for details) [11,12]. The benchmark execution was performed on all 17 protocols implemented in DockBench 1.0.6 based on seven different docking software options, each of which was coupled to different scoring functions whenever possible. The complete list of the protocols is reported in Table 1. The benchmark consisted of the execution of 70,873 single docking runs (4169 complexes; 17 protocols) distributed on a single server. The wall time necessary to perform all docking runs was approximately 72 h.

The automated analysis was based on the calculation of three scores: (i) RMSD minimum (RMSD_min_), (ii) the RMSD average (RMSD_ave_), (ii) the number of structure with RMSD lower than the (N_(RSMD < R)_), and a fourth score named Protocol Score P_score_ that summarized the overall performance for a geometric point of view. The P_score_ instead is defined as follows: One point is assigned to the protocols that have an RMSD_ave_ lower than the value of the crystallographic resolution, another point is assigned to the protocols producing at least 10 poses (50% of generated conformation) with an RMSD (compared to the crystallographic geometry) lower than the crystallographic resolution, and two points are assigned to protocols which fulfill both the previous conditions. The complete matrix of the results is available in supporting information. The observed RMSD_min_ values were in the range of 0.05 and 38.49 Å. High RMSD_min_ values are symptomatic for ligands placed far away from the native binding site. A possible explanation could be ascribed in having defined the pocket using a sphere with radius 15 Å. The radius was deliberately set large to give the possibility to be sufficiently broad for all the ligands in the dataset and may be problematic for docking of small ligands or in the case of multiple pockets closely located.

An interesting question we were considered was about the performance of docking protocols in different target families since, in PDBbind, many protein families are represented by several entries. The results were grouped on the basis of the protein in families (PF) using the Pfam (Protein Family) database families as definition [13]. For each complex, the PF Pfam code was retrieved for the protein chain and hence grouped. For many multi-domain proteins, a different Pfam code can be assigned depending on the domain solved in the structure; for instance, the proteins belonging to the family PF00069 (Protein Kinase) often contain domains labeled as PF02827 (Cyclic adenosine monophospate-dependent protein kinase inhibitor), PF00134 (Cyclin, N-terminal domain), PF02984 (Cyclin, C-terminal domain), and a few others. Some proteins cannot be classified in a single group, and therefore we merged those groups for analysis (for example, PF00183 and PF02518, Heat Shock Protein 90, HSP90 and GHKL domain). To address this issue, we compared the docking performance by the Protocol Score (P_score_) for the major cluster to investigate whether the docking performances of the different protocols vary among the different protein families.

Unexpectedly, the performance among different families showed a remarkable fluctuation (Table 2), with certain families having many protocols with P_score_ > 1 on most of the complexes. It is interesting to note that, between the best performing group (PF00104) and the worst (PF00026) one, the percentage of protocols with P_score_ > 1 showed a difference of an order of magnitude, 41.66%, and 4.37%, respectively. Among the best-performing ones, the families with good P_score_ were: PF00104 (Hormone receptors), PF00497 (Bacterial extracellular solute-binding proteins, family 3), PF10613 (Ligated ion channel l-glutamate and glycine binding site), and PF01048 (Phosphorylase superfamily). All these families showed a P_score_ > 1 in more than 29% of the docking runs.

On the other hand, we found that certain families had very poor results, with P_score_ > 1 found below 10%; this is the case for PF00194 (Eukaryotic-type carbonic anhydrase), PF00077 (Retroviral aspartyl proteases), PF00413 (Matrixin), and PF00026 (Eukaryotic aspartyl protease). The trend observed for P_score_ is also evident in RMSD_ave_.

**Figure 1:**
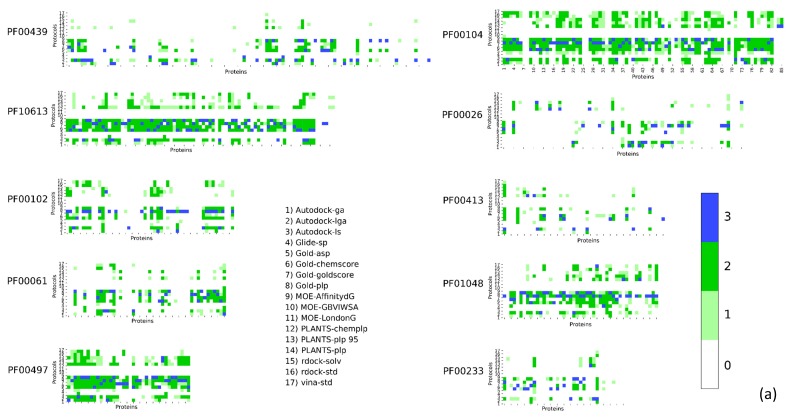

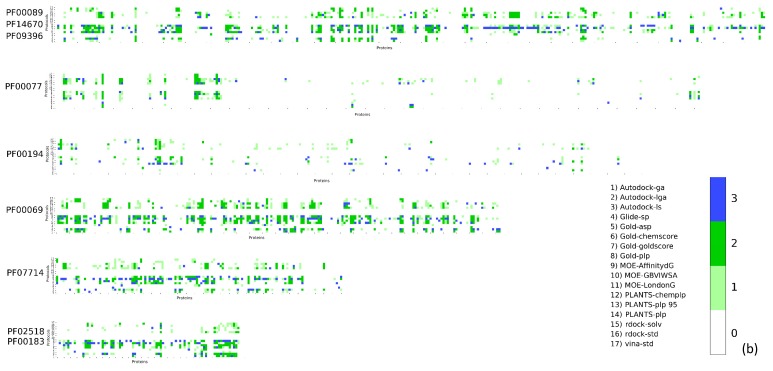
DockBench Results divided by Pfam protein families. The heatmaps are color-coded according to the P_score_. The ten families in panel (**a**) are: PF00439, Bromodomain; PF10613, Ligated ion channel l-glutamate and glycine-binding site; PF00102, Protein tyrosine phosphatases; PF000061 Lipocalin; PF00497, Bacterial extracellular solute-binding proteins family 3; PF00104, Hormone receptors; PF00026, Eukaryotic aspartyl protease Peptidase M_10; PF01048, Phosphorylase superfamily; PF00233, 3′5′-cyclic nucleotide phosphodiesterases. The six families in panel (**b**) are: PF00089 Trypsin, PF14670 Coagulation Factor Xa inhibitory site, PF09396 Thrombin light chain, PF00077 Retroviral aspartyl proteases, PF00194 carbonic anhydrases, PF00069 protein kinase, PF07714 tyrosine kinase, PF02518 GHKL domain, and PF00183 HSP90.

**Figure 2:**
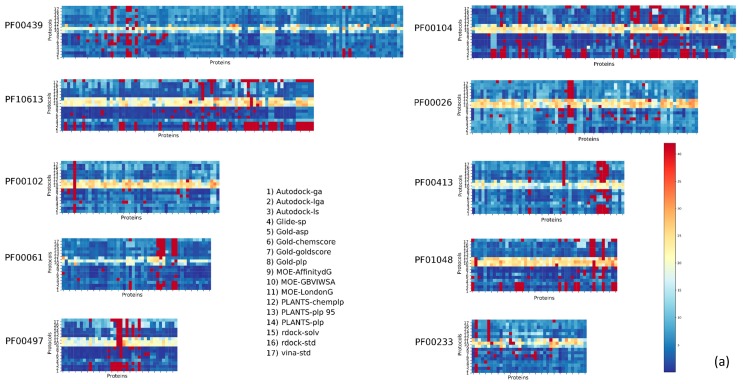

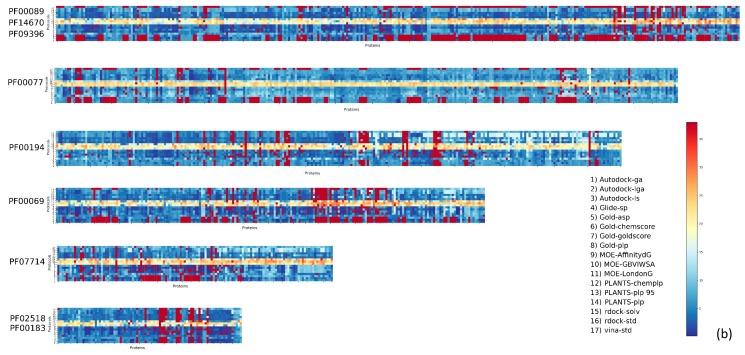
DockBench Results divided by Pfam protein families. The heatmaps are color-coded according to the RMSD_ave_. The ten families in panel (a) are: PF00439, Bromodomain; PF10613, Ligated ion channel l-glutamate and glycine-binding site; PF00102, Protein tyrosine phosphatases; PF000061 Lipocalin; PF00497, Bacterial extracellular solute-binding proteins family 3; PF00104, Hormone receptors; PF00026, Eukaryotic aspartyl protease Peptidase M_10; PF01048, Phosphorylase superfamily; and PF00233, 3′5′-cyclic nucleotide phosphodiesterases. In panel (**b**) the heatmaps are color-coded according to the Root Main Square Deviation (RMSD)_ave_. The six families are: PF00089 Trypsin, PF14670 Coagulation Factor Xa inhibitory site, PF09396 Thrombin light chain, PF00077 Retroviral aspartyl proteases, PF00194 carbonic anhydrases, PF00069 protein kinase, PF07714 tyrosine kinase, PF02518 GHKL domain, and PF00183 HSP90.

The results for the most populated families are reported in Figure 1. The P_score_s were reported as a heatmap to easily summarize the comparison of such a big matrix (higher scores highlight better protocol-complex couple). Numerical results are reported in the supplementary information. The results for the same families in terms of RMSD_ave_ are reported in Figure 2.

A further aspect that was considered was the ability of the docking protocol in placing in the first position, according to their scoring function, the pose with the lowest RMSD. This aspect is particularly relevant because it indicates how the protocol is able to distinguish between different binding modes and, hopefully, prioritizing a binding mode close to the experimentally observed. In Figures S1 and S2, the heatmap plots reporting for the docking runs in which the best-scored pose is also the conformation with lowest RMSD. Unfortunately, in several cases, this simultaneous occurrence did not always guarantee the identification od near-native pose. Indeed, we observed for several cases where the lowest RMSD conformation was far from the experimentally solved one with RMSD values reaching values bigger than 10 Å. The RMSD value of the best conformations is reported on the heatmaps in Figures S3 and S4. Therefore, we performed further analysis focusing on investigation of when the best pose also had a low RMSD value but not necessarily the lowest values. We decided to set a threshold of 1.5 Å to define a near-native pose. In this way, we could highlight a protocol able to place a “good” pose as the first solution, even if potentially better conformation could be present among the 20 obtained. In Figures S5 and S6, the runs that fulfill such concurrence are reported. Again, the performance of docking protocols showed a very different performance depending on the protein family and, interestingly, in agreement with the Pscore trends. The Ligand-binding domain of nuclear hormone receptor (PF00194) showed in 50% of the runs RMSD < 1.5 Å for the first pose. The percentage of success is also remarkable for the Ligated ion channel l-glutamate- and glycine-binding site (PF10613), 49.3%; the Bacterial extracellular solute-binding proteins (PF00497), 47.6%; and Phosphorylase superfamily (PF01048), 41.7%. On the contrary, certain families performed poorly in this analysis, in particular, Eukaryotic-type carbonic anhydrase, which showed only a 10.8% (Table S1, on Supporting Material).

**Figure 3:**
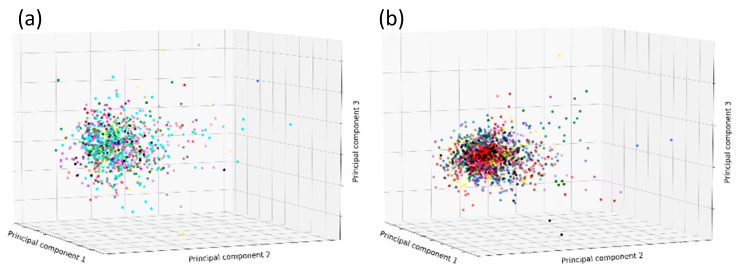
Principal Component Analysis (PCA) analysis seven molecular descriptors for the groups of ligands on the base of protein families in Table 2. The PCA analysis of ligands from the protein families were split into two groups according to the same division on Figure 1b (**a**) and Figure 2b (**b**). The descriptors used in the analysis are weight, rotatable bonds, hydrogen bond acceptor, hydrogen bond donor, clogP, total polar surface area, and van der Waals volume.

The factors that are so dramatically affecting the quality of the docking outputs among different families could be related to many variables. First, we address the possible different chemical natures of the ligands belonging to each protein family. To evaluate the ligand chemical space, several molecular descriptors were calculated, including weight, rotatable bonds, hydrogen bond acceptors, hydrogen bond donors, clogP, total polar surface area, and van der Waals volume. To reduce the number of the dimensions, and therefore make the distribution representable in a three-dimensional plot, a Principal Component Analysis (PCA) was performed. As can be seen in Figure 3, ligands of the different clusters do not seem to occupy a different portion of the chemical space. Hence, we then moved attention to possible players removed during the complex preparation, considering that the poor performances of docking in the cluster PF00077 (Retroviral aspartyl proteases) and PF00439 (Bromodomain) could be eventually ascribed to the removal of the crystallographic waters. It was already reported that the binding mode for several ligands is mediated by a series of water molecules for bromodomains [14].

Similarly, in the performances observed for cluster PF00194 (Carbonic Anhydrases), a crucial aspect could be represented by the removal of the zinc ion from the binding sites.

**Figure 4:**
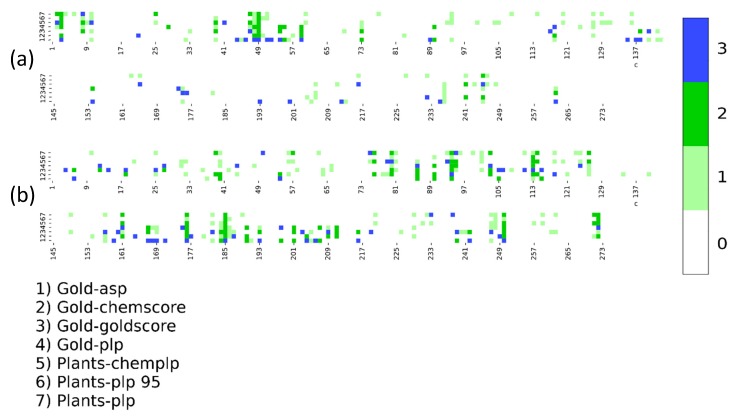
Comparison between DockBench Results in terms of Protocol Score for cluster PF00194 (carbonic anhydrases) with (**b**) and without (**a**) the Zinc ion.

For this reason, we performed a further benchmark focused on this family, including the Zinc ion, employing the most promising protocols in the first benchmark, Plants- and Gold-based protocols. The comparison of the heatmaps of the P_score_ reported in Figure 4 demonstrates that, despite the introduction of the Zinc ion, the trend of the P_score_ improves only moderately. Surprisingly, the distribution of the high P_score_ is different in the two benchmarks, suggesting that the Zinc ion introduction only improves for certain complex structures while getting worse for others.

## 3. Discussion

A computational chemist has to ask himself many of the right questions when facing molecular docking studies, and the answers are not univocal. Of course, the choice of the best performing protocol and, when multiple structures are available, of the target conformation is the most significant decision. However, the employment of molecular docking may have a different purpose, and a proficient protocol choice must consider such different use. If molecular docking is addressed in binding mode studies, the protocol performances should have the priority. At the same time, the choice of the protein target should depend on the similarity between the compounds to be studied and the ligand co-crystalized. When molecular docking is used in a VS campaign, more variables affect the selection, like the execution speed. The results obtained in this benchmark were obtained with parameter as close as possible to the default values resulting in very variable execution times. For instance, as already reported in previous Dockbench studies, certain protocols may require an order of magnitude of longer time in comparison to faster protocols. It is evident in the case of large libraries that this may represent a critical issue, hence protocols with similar outcomes in self-docking procedure where the choice can be influenced by the execution speed. In our benchmark, we observed, for example, in certain families of proteins, several protocols showing good performance, hence protocol selection may depend on the other factor. It is interesting to note that in the protein families in which molecular docking shows a good trend in reproducing the experimental conformation, certain protein–ligand complexes are far from being predicted correctly, suggesting the importance of excluding them for docking simulations. Differently, other protein families are challenging targets in which the choice of the posing-scoring algorithm seems to be crucial, as well as the identification of the most suitable complex structure. The performance of such a challenging target should also point out the necessity to investigate the issues that are affecting the docking calculation, for instance, in considering the role of stable water molecules in the binding site or the role of a cofactor, flexible regions of the pocket, or other drawbacks of the system. This study may help the user approach a new target by molecular docking in identifying promising protocols and excluding problematic complex structures. In our opinion, the assessment of the suitable procedure should become a good practice also in light of the increasing number of entries available in the PDB and the advent of novel techniques like Cryo-EM and Solid-State NMR are wading the landscape of an experimentally solved target.

## 4. Materials and Methods

### 4.1. Database Preparation

The Refined-set of the PDBbind database was obtained from PDBbind web service (http://www.pdbbind.org.cn/) [10]. This dataset is composed of 4463 protein–ligand complexes, and 4169 of them were used for this work. We excluded 294 structures containing peptide–protein complexes that are not particularly suitable for DockBench protocol since it used docking settings which were as close as possible to the default parameters provided by the developers of each software and mostly calibrated on small organic molecules typical of drug discovery.

These 4169 complexes were prepared as described below.

The protein structures have been prepared using a Scientific Vector Language (SVL) script using the functions contained in MOE suite [11] reproducing the protein preparation tool of MOE to fix crystal structures issues, such as prediction of coordinates of missing atoms of partially solved residues. Co-crystallized solvent molecules and impurities (such as co-solvents) were removed, and only protein and ligand coordinates were retained. For all ligands, the most favorable ionic state was calculated with OpenEye tools fixpKa [12]. The partial charges were assigned with molcharge, also part of OpenEye toolkit [12]. Ligand geometries were minimized in the first step of DockBench with Openbabel routing using the MMFF94 force field [15].

### 4.2. Benchmark: Software and Hardware

The benchmark was performed with DockBench 1.06 software [16,17], running on a single HP ProLiant server DL585G7, equipped with four AMD Opteron Processor 6282 servers, for a total of 64 CPU cores. Docking protocol was executed according to the original implementation already reported [16]. All the 17 protocols from seven different software options (AutoDock 4.2.5.1 [18], Vina 1.1.2 [19], PLANTS 1.2 [20], rDOCK [21], Glide 6.5 [22], Gold 5.4.1 [23,24], and MOE 2019.01 [11]) were included in the benchmark and run on all 4169 protein–ligand complexes. Briefly, 20 poses were generated every single run. The binding site was defined using a sphere having a radius of 15 Å centered on the center of mass of the co-crystalized ligand present in the complex. An RMSD threshold set to a value of 1 Å value to define unique poses.

The analysis was performed with DockBench analyzer coupled to external Python and Bash script to manage the notable amount of data and to produce the plots [25,26]. The Pfam Protein family was retrieved for each protein using the RCSD PDB REST API service [27], while the Pfam Clan was obtained from Pfam REST API service [13]. Molecular descriptors were calculated using MOE suite [11].

## Figures and Tables

**Table 1:** List of docking protocols used in the benchmark.

Program	Search Algorithm/Placing Method	Scoring Function	Protocol Abbreviation
Autodock 4.2	Local Search	AutoDock SF	AUTODOCK-ls
	Lamarckian GA	AutoDock SF	AUTODOCK-lga
	Genetic Algorithm	AutoDock SF	AUTODOCK-ga
Vina 1.1.2	Monte Carlo + BFGS local search	Standard Vina SF	VINA-std
Glide 6.5	Glide Algorithm	Standard Precision	GLIDE-sp
GOLD 5.4.1	Genetic Algorithm	Goldscore	GOLD-goldscore
	Genetic Algorithm	Chemscore	GOLD-chemscore
	Genetic Algorithm	ASP	GOLD-asp
	Genetic Algorithm	PLP	GOLD-plp
MOE 2019.01	Triangle Matcher	London-dG	MOE-londondg
	Triangle Matcher	Affinity-dG	MOE-affinitydg
	Triangle Matcher	GBIVIWSA	MOE-gbiviwsa
PLANTS 1.2	ACO Algorithm	PLP	PLANTS-plp
	ACO Algorithm	PLP95	PLANTS-plp95
	ACO Algorithm	ChemPLP	PLANTS-chemplp
rDock 2013.1	Genetic Algorithm + Monte Carlo + Simplex minimization	Standard rDock master SF	RDOCK-std
	Genetic Algorithm + Monte Carlo + Simplex minimization	Standard rDock master SF + desolvation potential	RDOCK-solv

GA (Genetic Algorithm) BFGS (Broyden-Fletcher-Goldfarb-Shanno), ASP (Astex Statistical Potential), PLP (pair wise linear potential), ACO (Ant Colony Optimization).

**Table 2:** Summary of benchmark results by Pfam families. Protocol scores are reported as percentage with respect to the total docking runs (P_score_%).

Pfam Family	Protein Description	Size	Protocol Score P_score_%				
			0	1	2	3	>1
PF00104	Ligand-binding domain of nuclear hormone receptor	85	59.34	10.24	26.57	4.84	41.66
PF00497	Bacterial extracellular solute-binding proteins, family 3	38	59.29	9.44	25.70	5.57	40.71
PF10613	Ligated ion channel l-glutamate- and glycine-binding site	83	67.97	6.80	20.55	4.68	32.03
PF01048	Phosphorylase superfamily	47	70.09	8.01	16.77	5.13	29.91
PF00102	Protein-tyrosine phosphatase	52	79.30	5.20	11.99	3.51	20.70
PF00069	Protein kinase domain	207	80.68	5.43	10.46	3.43	19.32
PF00061	Lipocalin/cytosolic fatty-acid binding protein family	49	82.11	4.08	10.80	3.00	17.88
PF02518 PF00183	Hsp90 protein and GHKL domain	89	82.74	5.35	8.26	3.64	17.25
PF07714	Protein tyrosine kinase	133	83.90	5.79	6.77	3.54	16.10
PF00089 PF14670 PF09396	Trypsin	330	85.54	4.65	6.84	2.96	14.45
PF00233	3′5′-cyclic nucleotide phosphodiesterase	37	87.92	3.82	5.41	2.86	12,08
PF00439	Bromodomain	112	90.02	2.89	4.67	2.42	9.98
PF00026	Eukaryotic aspartyl protease	73	90.49	3.14	4.11	2.26	9.51
PF00413	Matrixin	49	90.88	3.24	4.20	1.68	9.12
PF00077	Retroviral aspartyl protease	301	95.41	2.27	1.64	0.68	4.59
PF00194	Eukaryotic-type carbonic anhydrase	273	95.63	2.28	1.17	0.91	4.37

Pfam (Protein Family), Hsp90 (Heat shock protein 90).

## Supplementary Materials

The complete table of results is provided as excel file (SI_DockBenck_resuld.xls). Supplementary materials can be found at https://www.mdpi.com/1422-0067/20/14/3558/s1.

## Abbreviations

SB	Structure-Based
VS	Virtual Screening
PFAM	Protein Family
NMR	Nuclear Magnetic Resona
CryoEM	Cryo Electron Microscpy
MMFF94	Merck Molecular Force Field
RMSD	Root Main Square Deviation
