# Supplementary material for: Can We Still Trust Docking Results? An Extension of the Applicability of DockBench on PDBbind Database
Source: Int J Mol Sci. 2019 Jul 20;20(14):3558. doi: 10.3390/ijms20143558 (PMC6679043; doi:10.3390/ijms20143558)
Supplement: Supplementary file 1 [file ijms-20-03558-s001.zip › ijms-540125-supplementary.pdf]

Can we still trust docking results? An extension of the applicability of DockBench on PDBbind database.

Giovanni Bolcato [1], Alberto Cuzzolin [1], Maicol Bissaro [1], Stefano Moro[1,*] and Mattia Sturlese [1,*]

[1] Molecular Modeling Section, Department of Pharmaceutical and Pharmacological Sciences, University of Padova, 35131 Padova, Italy.
[*] Correspondence: stefano.moro@unipd.it; mattia.sturlese@unipd.it

Supplementary Materials

Table of content

**Figure S1**	Docking runs having as first pose the lowest RMSD conformation: Heatmaps of most populated protein families (first part)	Page 2
**Figure S2**	Docking runs having as first pose the lowest RMSD conformation: Heatmaps of most populated protein families (second part)	Page 3
**Figure S3**	Plots reporting the RMSD of the first pose: Heatmaps of most populated protein families (first part)	Page 4
**Figure S4**	Plots reporting the RMSD of the first pose: Heatmaps of most populated protein families (second part)	Page 5
**Figure S5**	Docking runs having the first pose characterized by an RMSD value lower than 1.5 Å: Heatmaps of most populated protein families (first part)	Page 6
**Figure S6**	Docking runs having the first pose characterized by an RMSD value lower than 1.5 Å: Heatmaps of most populated protein families (second part)	Page 7
**Table S1**	Percentage of runs with native-like conformation reported as the first pose	Page 8

Figure S1

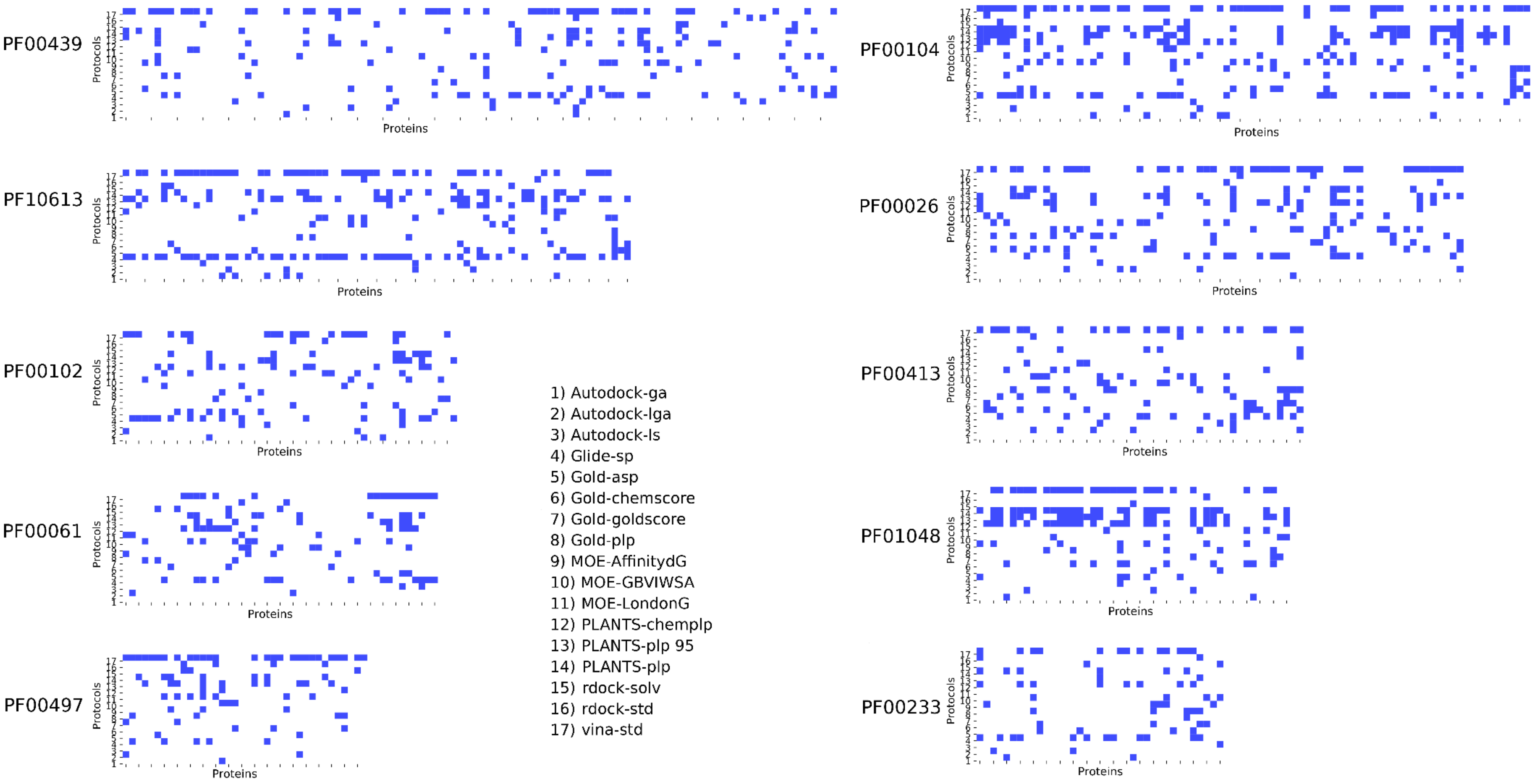

Figure S1. DockBench Results divided by Pfam protein families. The heatmaps show in blue the cases in which the docking run reported at best solution the conformation with the lowest RMSD among the 20 generated. The ten families are: PF00439, Bromodomain; PF10613, Ligated ion channel L-glutamate and glycine-binding site; PF00102, Protein tyrosine phosphatases; PF000061 Lipocalin; PF00497, Bacterial extracellular solute-binding proteins family 3; PF00104, Hormone receptors; PF00026, Eukaryotic aspartyl protease Peptidase M_10; PF01048, Phosphorylase superfamily; PF00233, 3'5'-cyclic nucleotide phosphodiesterases.

Figure S2

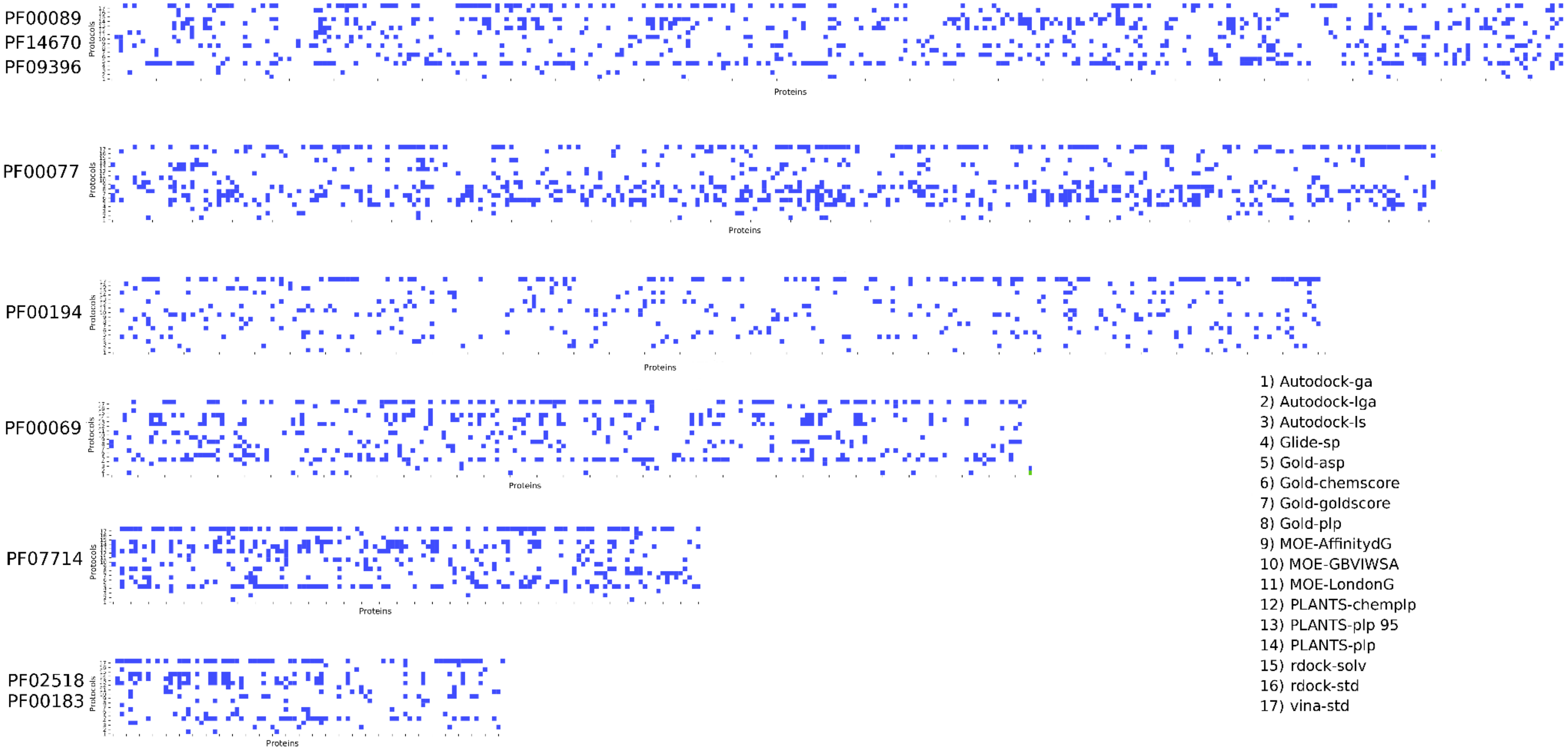

Figure S2. DockBench Results divided by Pfam protein families. The heatmaps show in blue the cases in which the docking run reported at best solution the conformation with the lowest RMSD among the 20 generated (continued from figure S1). The six families are PF00089 Trypsin, PF14670 Coagulation Factor Xa inhibitory site, PF09396 Thrombin light chain, PF00077 Retroviral aspartyl proteases, PF00194 carbonic anhydrases, PF00069 protein kinase, PF07714 tyrosine kinase, PF02518 GHKL domain, PF00183 HSP90.

Figure S3

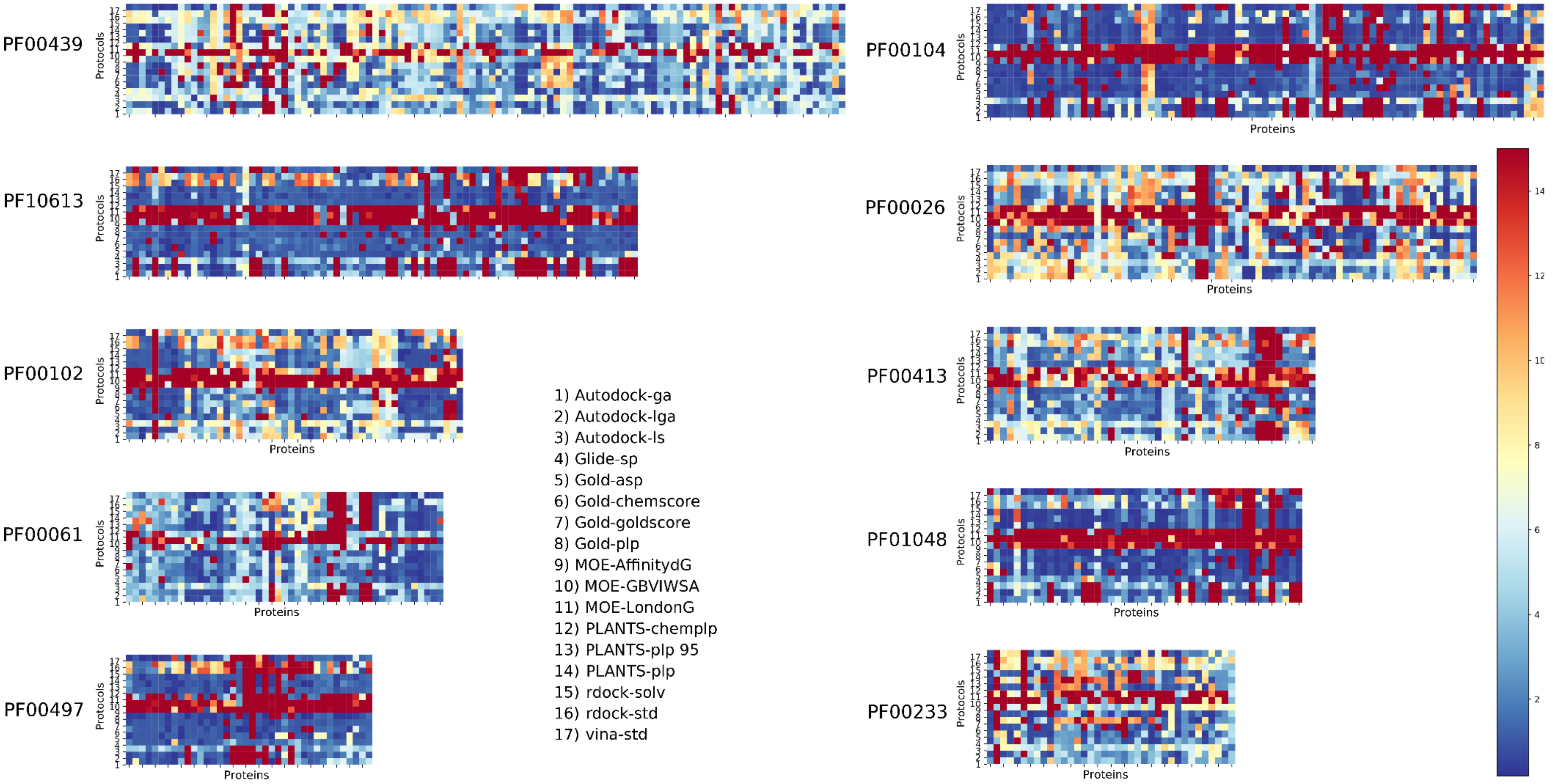

Figure S3. DockBench Results divided by Pfam protein families. The heatmaps RMSD of the best solution, according to the scoring function, among the 20 generated. The ten families are: PF00439, Bromodomain; PF10613, Ligated ion channel L-glutamate and glycine-binding site; PF00102, Protein tyrosine phosphatases; PF000061 Lipocalin; PF00497, Bacterial extracellular solute-binding proteins family 3; PF00104, Hormone receptors; PF00026, Eukaryotic aspartyl protease Peptidase M_10; PF01048, Phosphorylase superfamily; PF00233, 3'5'-cyclic nucleotide phosphodiesterases.

Figure S4

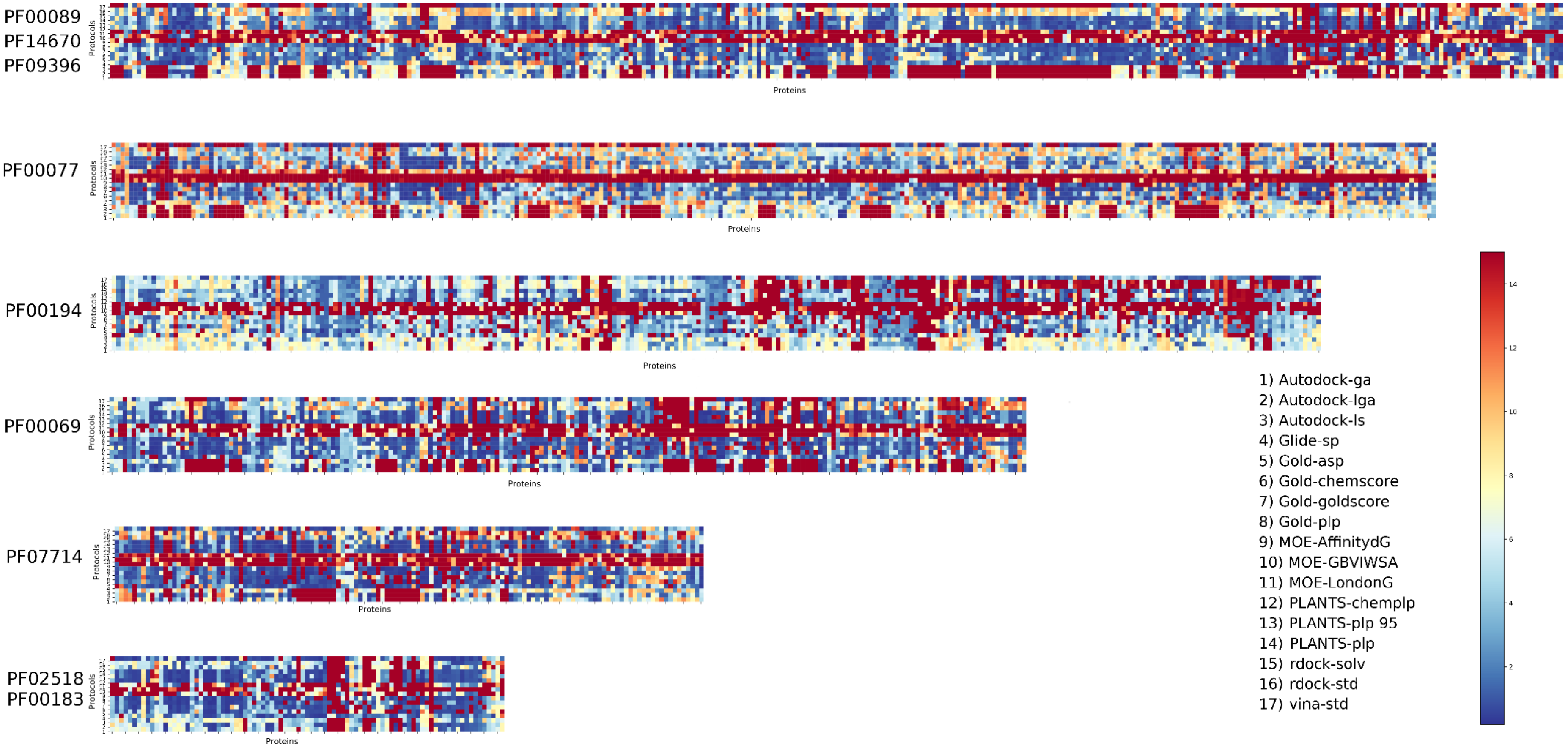

Figure S4. DockBench Results divided by Pfam protein families. The heatmaps RMSD of the best solution, according to the scoring function, among the 20 generated (continued from figure S3). The six families are PF00089 Trypsin, PF14670 Coagulation Factor Xa inhibitory site, PF09396 Thrombin light chain, PF00077 Retroviral aspartyl proteases, PF00194 carbonic anhydrases, PF00069 protein kinase, PF07714 tyrosine kinase, PF02518 GHKL domain, PF00183 HSP90.

Figure S5

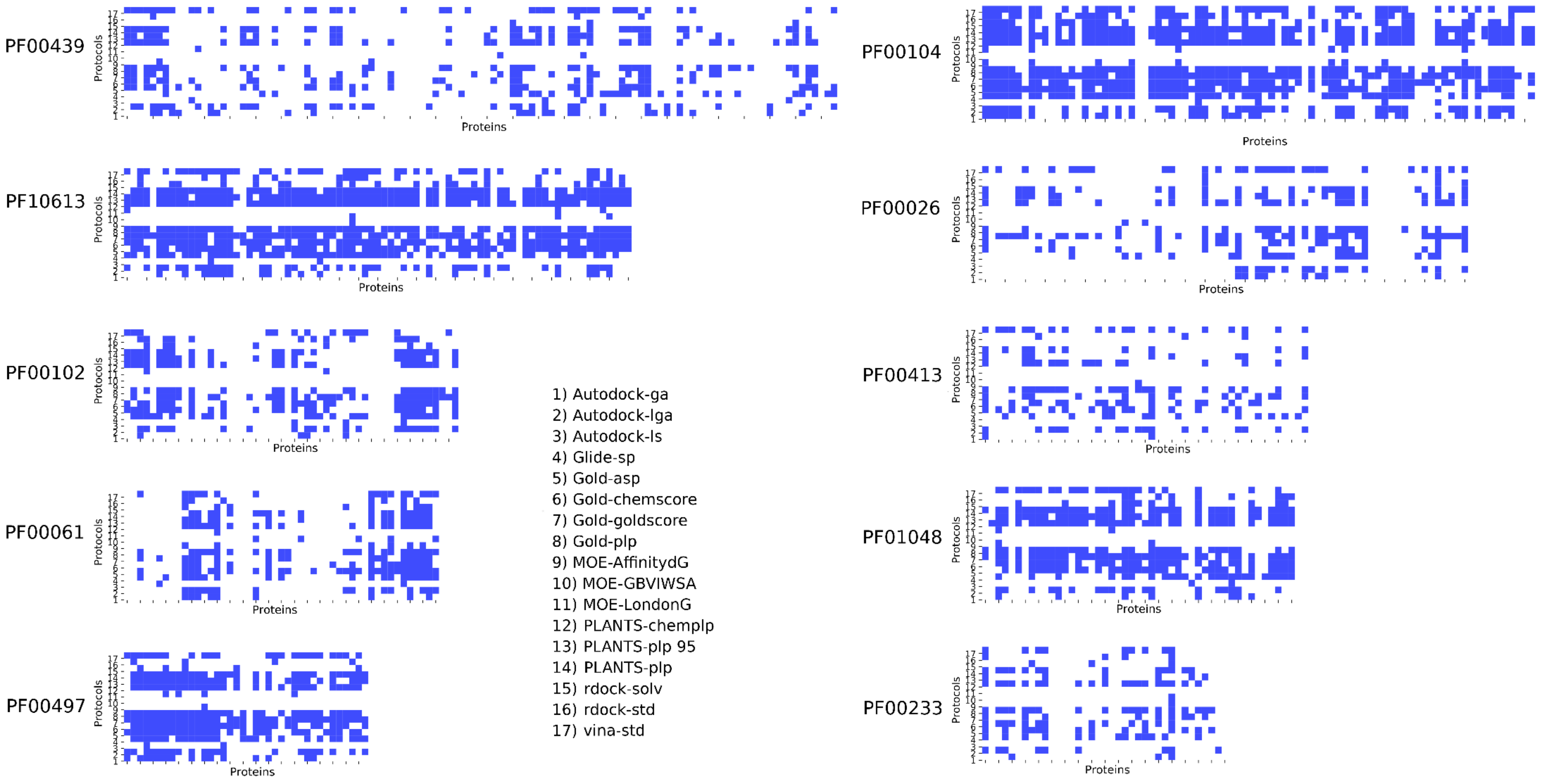

Figure S5. DockBench Results divided by Pfam protein families. The heatmaps show in blue the cases in which the docking run reported at best solution a conformation having an RMSD lower than 1.5 Å. The ten families are: PF00439, Bromodomain; PF10613, Ligated ion channel L-glutamate and glycine-binding

site; PF00102, Protein tyrosine phosphatases; PF000061 Lipocalin; PF00497, Bacterial extracellular solute-binding proteins family 3; PF00104, Hormone receptors; PF00026, Eukaryotic aspartyl protease Peptidase M_10; PF01048, Phosphorylase superfamily; PF00233, 3'5'-cyclic nucleotide phosphodiesterases.

Figure S6

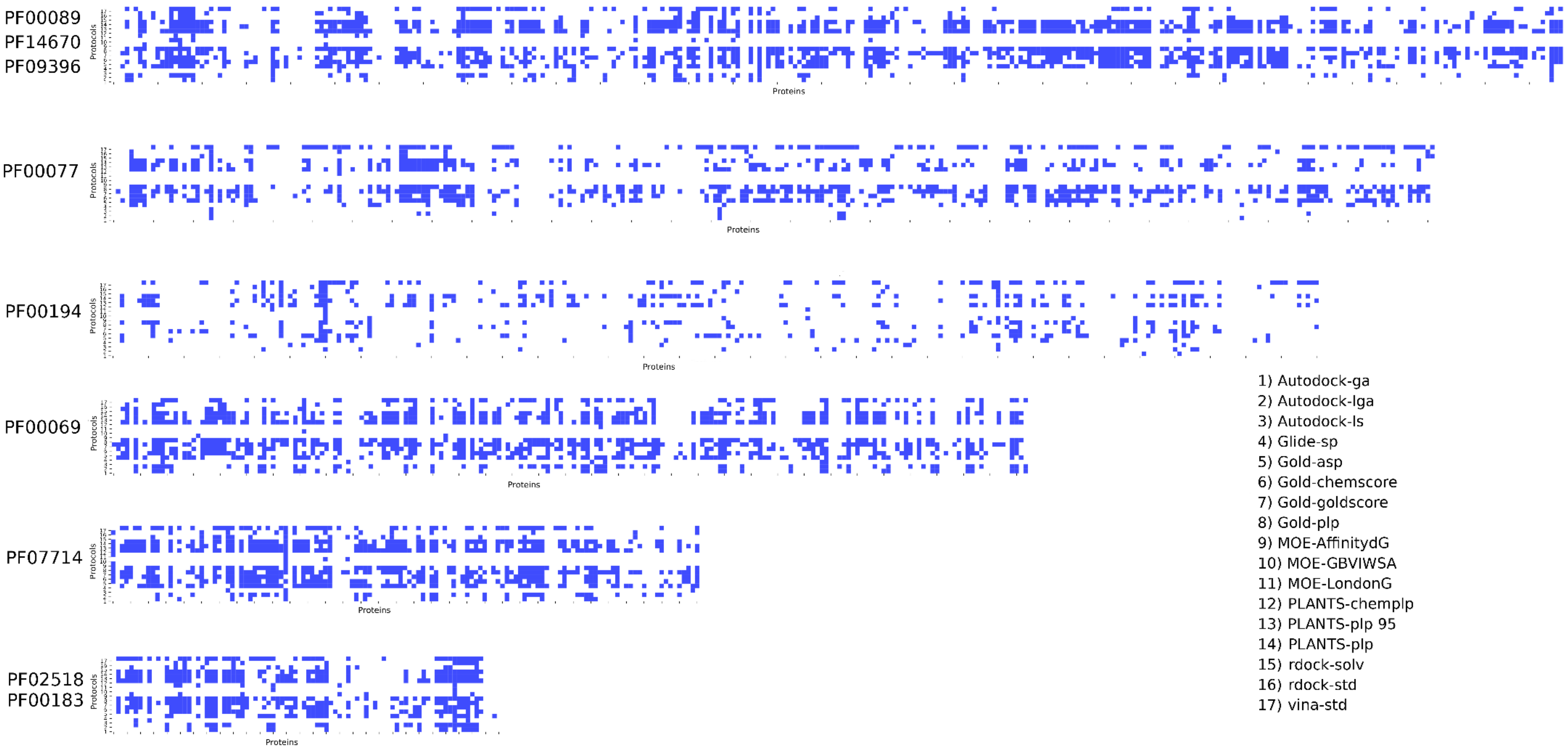

Figure S6. DockBench Results divided by Pfam protein families. The heatmaps show in blue the cases in which the docking run reported at best solution a conformation having an RMSD lower than 1.5 Å (continued from figure S5). The six families are PF00089 Trypsin, PF14670 Coagulation Factor Xa inhibitory site, PF09396 Thrombin light chain, PF00077 Retroviral aspartyl proteases, PF00194 carbonic anhydrases, PF00069 protein kinase, PF07714 tyrosine kinase, PF02518 GHKL domain, PF00183 HSP90.

Table S1

Pfam Family	Protein Description	% of runs with the first poses with RMSD < 1.5 Å
PF00104	Ligand-binding domain of nuclear hormone receptor	50.42
PF00497	Bacterial extracellular solute-binding proteins, family 3	47.60
PF10613	Ligated ion channel L-glutamate- and glycine-binding site	49.33
PF01048	Phosphorylase superfamily	41.75
PF00102	Protein-tyrosine phosphatase	28.93
PF00069	Protein kinase domain	29.15
PF00061	Lipocalin / cytosolic fatty-acid binding protein family	27.21
PF02518<br>PF00183	Hsp90 protein	31.84
PF07714	Protein tyrosine kinase	31.79
PF00089<br>PF14670<br>PF09396	Trypsin	27.99
PF00233	3'5'-cyclic nucleotide phosphodiesterase	21.75
PF00439	Bromodomain	19.60
PF00026	Eukaryotic aspartyl protease	19.57
PF00413	Matrixin	19.06
PF00077	Retroviral aspartyl protease	18.78
PF00194	Eukaryotic-type carbonic anhydrase	10.84

Table S1. The percentage within the protein family of docking runs having successfully fulfilled the criteria of placing as first pose a conformation with RMSD value lower than 1.5 Å.
